# Nutritional and Environmental Influences on Athlete Health and Performance

**DOI:** 10.1007/s40279-018-0863-y

**Published:** 2018-01-24

**Authors:** Lawrence L. Spriet

**Affiliations:** Human Health and Nutritional Sciences, University of Guelph, Guelph, ON N1G 2W1 Canada

Athletes continually push themselves to achieve improvements in performance and success in personal and competitive athletic situations. Many researchers are actively engaged in investigating approaches to improve ‘real-world’ athletic performance and this extends far beyond traditional laboratory-based testing and experimentation. These applied researchers realize that to maximize the potential for success, what happens outside of training and competition situations can have a major impact on performance. The concept of ‘staying healthy’ or ‘optimizing health’ is paramount for maintaining a stable environment where the athlete can engage in fruitful training sessions and successful competitions.

The Gatorade Sports Science Institute (GSSI) has been bringing basic and applied sports nutrition researchers together for the past 5 years to address many issues that relate to the health and success of athletes. This continued in 2016 with a meeting held in October to discuss several nutritional and environmental issues that influence athlete health and performance. Following the meeting, the authors summarized the recent work in their topic area, resulting in the articles in this Sports Medicine supplement. This collection of papers is the fifth in a series of GSSI-supported Sports Medicine supplements.

The first paper addresses whether athletes need to supplement with vitamin D. This topic is controversial as a clear understanding of what to measure in blood samples is far from decided and results vary as a function of ethnicity. Future testing for vitamin D status will need to address the bioavailable or ‘free’ vitamin D levels in athletic groups to permit new and accurate thresholds and target concentrations to be established. The next two papers in the supplement examine the value of drinking during exercise. One paper examines the potential of ingesting cold water/ice slurries to cool the body during endurance exercise in the heat. While it seems intuitive that this would be effective, there is a compensatory decrease in sweating that negates any cooling advantage in many exercise situations. The second paper addresses the much-debated topic of how much an athlete should drink during exercise to maintain or improve performance. The paper addresses ‘planned drinking’ versus ‘drinking to thirst’ and outlines when these approaches to maintaining hydration are appropriate.

The final four papers of the supplement address the roles that nutrition may play in minimizing injury, remodeling skeletal muscle, maintaining health, and improving performance. Sports-related head trauma is a topic that concerns many athletes, coaches and athletic support personnel in today’s sporting culture. Attempts at protecting the head from injury with education, rule changes and equipment have not yet been fully successful and nutritional supplementation may help minimize the effects of head trauma and/or speed recovery from these injuries. Remodeling of skeletal muscle is an important and ongoing process with highly active athletes. While methods and mechanisms of stimulating muscle protein synthesis have been widely studied, muscle protein breakdown has received less attention, even though it is important for muscle remodeling, adaptation to training, and increasing muscle mass. The paper devoted to this topic concludes that little is known about the effects of nutrition on muscle protein breakdown and that new methods are needed to gain insight in this area. Nutrition also plays a very important role in maintaining the health of athletes, and this is especially true in the areas of respiratory and gut health, and mucosal immunity. While upper respiratory symptoms are the most common illness in athletes, nutrition can have a positive effect on the microbiota of the nose, mouth, respiratory tract and gut to ultimately strengthen mucosal immunity. Lastly, moderate doses of caffeine delivered in capsules and coffee improve performance in a wide variety of athletic and sporting situations for athletes of all levels. With the realization that low doses of caffeine are also ergogenic, the administration of caffeine in a variety of alternate forms has emerged. This includes chewing gums, gels, bars, lozenges, aerosols, etc., with varying success in delivering caffeine to critical locations in the body and improving athletic performance.

A common theme in the papers of this supplement examining the nutritional and environmental influences that may affect the health and performance of athletes is that a great deal of additional research is needed! Hopefully, these papers will stimulate thinking and excite sports nutrition researchers to conduct additional research in these areas in the years to come.

Lawrence L. Spriet, PhD

Guest Editor

This article was published in a supplement supported by the GSSI. The supplement was guest edited by Lawrence L. Spriet, who attended a meeting of the GSSI Expert Panel in October 2016 and received honoraria from the GSSI, a division of PepsiCo, Inc., for his participation in the meeting and the writing of a manuscript. He received no honoraria for guest editing the supplement. Dr. Spriet selected peer reviewers for each paper and managed the process, except for his own paper.

